# The Doctor's Difflculties in Town and Country

**Published:** 1891-02-28

**Authors:** 


### The Hospital

A Weekly Journal of

**Science, Medicine, Nursing, and Philanthropy.**

*For Hospitals, Asylums, Infirmaries, Dispensaries, Medical Practitioners, Students, Nurses, and the Charitable Public.*

Vol. IX.—No. 231.] [February 28, 1891.

### THE DOCTOR'S DIFFICULTIES IN TOWN AND COUNTRY.

It is a good many years ago now since A. K. H. B. gave to the world his "Recreations of a Country Parson." The title was novel, and the "recreations" extremely innocent. The book, moreover, was one long but very pleasant gossip; and so it had a great run, not only among the parsons to whom it specially appealed, but also among that large and gossip-loving class who circle around the parson as bees do round the maternal hive. Quite lately that vivacious East country rector, the Rev. Dr. Augustus Jessop, has issued "The Trials of a Country Parson." Dr. Jessop is assured of a host of readers, not only because he knows by experience the trials of a country parson as well as any man in England, but also because he can set them forth in due order; and still better, can teach his readers all the surest and most quickly operative arts of minimising their trials, and, when that is possible, of driving them quite away.

Writers write about parsons, and parsons write about themselves; but as yet nobody, not even a doctor, has taken in hand to present the public with any private and personal information about doctors. Perhaps, as a class, medical men cannot lay claim to being anything like so interesting as the clergy. Time was when no novel was complete without its curate; and that adventurous and heroic croquet player was not seldom the chief dish at the intellectual feast. It must be admitted that those days have gone by. The curate is no longer a social hero. If anything, he is beginning to be shunned, both by fathers with marriageable daughters and by the very prudent daughters themselves. The curate is going out: but is the young doctor coming in? We very much doubt it! The truth is that young professional men are for the most part the possessors of "lean and hungry" incomes; and in these times the very last thing that young ladies favour is anything that comes within a hundred miles of leanness and hunger of any sort. But in spite of these various facts and per contras doctors are a decidedly interesting class, at any rate to themselves. There is not lacking, also, a good deal of evidence which goes to show that they are becoming much more interesting to other people than were some former generations of their order. It would be strange if it were otherwise in this science-loving age. For it is a science-loving age, whether it be scientific or not; and doctors are first and above all things desirous of being, and of being considered, true "men of science."

The doctor certainly has "difficulties" both in town and country; and difficulties which much concern his patients. Perhaps the very chiefest of all the town doctor's difficulties is that town air, town work, town worries, and town life generally, prevent him from curing his patients so quickly and thoroughly as he would desire, and from keeping them well when they are well. Let no cynic say that the doctor, when once a patient is on his list, is seldom in a very great hurry to cure. That is an entire mistake. The ordinary town doctor is most anxious to cure his patients quickly, and for this reason, even if for no other, that it pays him to do so. The town man, and even the town woman and the town child, can never afford to be long ill. Business, and evermore business, calls the man with a loud and brazen tongue to get up from his bed and hasten to his office. Family cares or social claims pester the mother with equal pertinacity; whilst school—school where high fees are paid, where teachers are exigeant, where competition is keen and urgent—school draws the young from hours of sickness with a fascination which they find irresistible. In sickness, therefore, when the sickness is in a large town, the doctor must cure and cure quickly. If he does not, he is asked to stand aside and allow somebody else to try.

But it is exactly in the largest towns that quick curing is the most difficult. Town air is always impure and often poisonous. Witness the miserable fogs, accompanied by cold east winds, of last week. The doctor has a patient with double pneumonia—he is a man of forty-five: he will not believe that a serious illness is coming upon him, and goes to his office to the very last minute. He takes to his bed, and is poulticed or otherwise actively treated for a few days; the crisis seems to be passing, the breathing becomes easier, and the temperature lower; hope fills every heart. Suddenly the whole town is enveloped in a thick, raw, choking, and death-bringing fog: the patient cannot breathe, the temperature rushes up again, fresh blood appears in the expectoration, despair seizes upon the sick man, the household, and almost upon the doctor too. Whose fault was it? It was the fault of the deadly fog. In two or three weeks the patient is convalescent. Then he needs the pure breezes of the sea. If he cannot have those it is imperative that he should have a change of some kind. The atmosphere of the sick room is fatal to him. But he is in London; the house is small and the family large; he cannot have even a fresh bed-room. To open the window may not be to let in fresh and pure breezes, but cold and chilling blasts full of deadly poison. How is the convalescent to be restored quickly? How is the needed strength to be brought back with promptitude? How is the expectoration to be dried up? It cannot be done, at any rate in the unvitalising atmosphere of the town. The sorrow to the family, as well as the loss and the injury, may be very great. But they may be almost as great to the doctor, unless the patient and his friends are unusually discriminating and just in their appreciation of the facts.

This is but one example of the difficulties of the doctor in a large town. Other cases will occur at once to the professional mind. Anæmic women, strumous children, dyspeptic men of business, sleepless stockbrokers—all these and innumerable like instances present to the doctor a *menu* of anxious daily toil which nobody need wish to share with him. When to all this is added the fact that competition is so keen and eager that patients will sometimes kick at even a second consultation, and that they know so little of medicine that they cannot form any kind of just opinion either of their own condition or of the wisdom of the methods employed to relieve it, there is, we think, in the town doctor's lot, something which claims the sympathy and interest of all intelligent and kindly-disposed men.

The difficulties of the country practitioner are quite of another kind, and are, on the whole, much less harassing and urgent. But this, we submit, is a difficulty, and not a very pleasant one:—to ride five miles at two o'clock on a winter morning to a farmhouse, over rough roads and uplands, where the east wind takes your nose off; to find the farmer groaning with a colic which a single ounce of castor oil would have cured, and which he need not have had at all if he had not eaten too much cold beef, pickled cabbage, and cheese for his supper; to ride home again over the same cold five miles—ten miles in all; to charge ten shillings for your night's work; to send in your account at Christmas, and to find that the farmer thinks you have charged too much; to give him credit for a couple of years, and then to be glad to settle the business for a payment of seven-and-sixpence. Is that a difficulty or is it not?

But what harasses and disappoints the country doctor most of all is his want of leisure for reading and for advancing with the progress of the times. He will tell you pathetically that he was a "student" at college, but that he is not a student now. He has a newly-qualified assistant, and he is ashamed to talk to him for fear of exposing his own ignorance. The young man himself is too inexperienced and foolish to have any shame at all; and so he assumes airs of superiority over his senior, who at the bedside is worth a whole regiment of such callow goslings. Then he has, perhaps, a terrible neighbour, the only other practitioner in the place. This *confrère*, who is too often an opponent and a rival, is not a gentleman. He will not keep to his own side of the fence, and shoot on his own preserves. He is a poacher, and carries an air gun. He is vicious and spiteful: he does not stick at a lie when occasion serves. To be at peace with him is impossible the miserable years become one long strife with a man who is only fit to stand up in the weekly market and sell pills by Dutch auction. These, and many like them, are the country doctor's difficulties. If he be a man, he fights them down and makes the best of them. But since he is a man, and often a man of culture, it is neither an easy nor a congenial lot which has fallen to his share. Let him be understood and intelligently sympathised with: it is all he asks.

